# The abscopal effect of local radiotherapy is induced by TGFβ blockade

**DOI:** 10.1186/2051-1426-1-S1-P115

**Published:** 2013-11-07

**Authors:** Julie Diamond, Claire Vanpouille-Box, Mary Helen Barcellos-Hoff, Silvia C Formenti, Sandra Demaria

**Affiliations:** 1 Pathology, NYU School of Medicine, New York, NY, USA; 2 Radiation Oncology, NYU School of Medicine, New York, NY, USA

## 

Radiotherapy (RT) is employed to achieve local cancer control. However, in rare patients, regression of metastases outside of the radiation field has been reported after irradiation of one tumor site, a phenomenon known as abscopal effect. We have previously shown in experimental tumor models that the abscopal effect is mediated by activation of anti-tumor immune responses by radiotherapy, which can convert the irradiated tumor into an in situ vaccine. However, effective induction of anti-tumor immunity by radiation is rare. To study the barriers to the induction of an abscopal effect we have employed a mouse model of metastatic breast cancer. Ionizing radiation activates Transforming Growth Factor-β (TGFβ), a strongly immunosuppressive cytokine that promotes DNA damage repair (DDR) and metastasis. We therefore hypothesized that neutralization of TGFβ may improve the development of anti-tumor immune responses induced by RT, leading to an abscopal effect. To test this hypothesis, mouse mammary carcinoma TSA cells were injected s.c. at day 0 into syngeneic immunocompetent BALC/c mice at two separate sites, a “primary” site that was irradiated, and a secondary site outside of the radiation field. TGFβ neutralizing 1D11 mAb was given i.p. starting one day before radiation. On day 12 when both tumors were palpable, mice were randomly assigned to groups receiving either 1D11 mAb or isotype control (MOPC-21) every other day for 16 days, with or without radiation (6 Gy doses given to the primary tumor on days 13-17). Radiation alone effectively delayed primary but not secondary tumor growth. 1D11 alone did not have a significant effect on either primary or secondary tumors. Combination of 1D11 with RT enhanced significantly inhibition of the primary irradiated tumor, with complete tumor regression in 4 out of 6 mice by day 28 (p=0.0059 radiation+1D11 versus radiation). Importantly, an abscopal effect was seen only in mice treated with radiation + 1D11, with significantly smaller secondary tumors on day 28 (p=0.0329 radiation+1D11 versus MOPC-21). Data indicate that blocking TGFβ in the context of radiation not only improves local tumor control, but also induces an abscopal effect with systemic tumor inhibition. Overall, data provide further support for the use of agents targeting TGFβ during radiotherapy, a concept currently tested in a phase I/II clinical trial in metastatic breast cancer patients (NCT01401062).

